# A Study on *Catalase* Gene Promoter Polymorphism *-21 A/T (rs7943316)* in Healthy Pakistani population

**DOI:** 10.12669/pjms.336.13188

**Published:** 2017

**Authors:** Syeda Nuzhat Nawab, Sitwat Zehra, Asher Fawwad, Abid Azhar

**Affiliations:** 1 Dr. Syeda Nuzhat Nawab, Scientific Officer, The Karachi Institute of Biotechnology and Genetic Engineering (KIBGE), University Of Karachi, Karachi, Pakistan; 2 Sitwat Zehra, PhD. Associate Professor, The Karachi Institute of Biotechnology and Genetic Engineering (KIBGE), University Of Karachi, Karachi, Pakistan; 3 Asher Fawwad, PhD, Associate Professor, Department of Biochemistry, Baqai Medical University, Senior Research Scientist, Research Department, Baqai Institute of Diabetology and Endocrinology, Karachi, Pakistan; 4 Abid Azhar, PhD.Director General, The Karachi Institute of Biotechnology and Genetic Engineering (KIBGE), University Of Karachi, Karachi, Pakistan

**Keywords:** Antioxidant enzyme, Catalase, Polymorphism, Promoter, Reactive oxygen species

## Abstract

**Background & Objective:**

Catalase (CAT) is an important endogenous antioxidant enzyme that detoxifies H_2_O_2_ into water and oxygen, consequently limiting the deleterious effects of reactive oxygen species. It has suggested that *CAT-21A/T (rs7943316)* OMIM: *115500* gene promoter polymorphism is predominantly associated with different human disorders such as hypertension, cancers, diabetes, nephropathy, and other diseases accompanied by oxidative stress. This study was designed to investigate the prevalence of mutant T allele frequency in healthy individuals.

**Methods:**

The study group consisted of 110 healthy individuals were enrolled from Baqai Institute of Diabetology and Endocrinology (BIDE), Karachi, Pakistan, during the period of April 2010 to May 2013. DNA was isolated from leukocytes. Genotyping of *CAT-21A/T (rs7943316)* gene promoter polymorphism was carried out using thermal cycler followed by RFLP. Blast N analysis was performed for the confirmation of gene sequences.

**Results:**

In *CAT-21A/T (rs7943316)* gene promoter polymorphism, wild type genotype (AA) was observed in 18.26% and alterered genotype (AT/TT) found in 81.74% cases.

**Conclusions:**

Data demonstrates that frequency and distribution of mutant T allele was more prevalent as compared to wild type A allele in the study group.

## INTRODUCTION

Reactive species of oxygen may cause oxidative stress which has been shown to play a major role in the pathogenesis of many diseases such as cancer, hyperlipidemia, diabetes mellitus, metabolic disorders, atherosclerosis, cardiovascular diseases (hypertension, ischemic heart disease, chronic heart failure), and neurodegenerative diseases.^1^ Most of the antioxidant enzyme genes have susceptibility for polymorphism and may contribute in the alteration of gene expressions and decrease the activity of these enzymes.^2^ Different metabolic disorders are shown to be associated with the modified antioxidant enzymes functions.^3^ Catalase (CAT) is one of the powerful antioxidant enzymes because of its highest turnover rate and exists in almost all aerobically respiring organisms.^4^

CAT has the ability to control oxidative stress by the decomposition of hydrogen peroxide (H_2_O_2_) into two molecules of water and one molecule of oxygen. Studies have mainly focused on the genetic polymorphisms of *CAT* gene which exist in coding and non coding regions and less attention has been paid to promoter region polymorphisms. *CAT -21A/T (rs7943316)* gene polymorphism in promoter region may change the binding affinity of transcription factors. Due to the presence of mutant T allele, inappropriate transcription factors binding may lead to altered promoter activity, gene expression and diminished catalytic activity of the enzyme. This reduced antioxidant enzyme catalytic activity may increase the susceptibility of oxidative stress and its related disorders.^3^-^6^ This study was designed to focus on the genetic variant *-21A/T (rs7943316)* of the *CAT* gene and prevalence of mutant T allele frequency in healthy individuals.

## METHODS

### Study Design

After approval of study from the ethical review board committees of respective institutions, one hundred and ten (110) healthy subjects were enrolled from Baqai Institute of Diabetology and Endocrinology (BIDE), Karachi, Pakistan, during the period of April 2010 to May 2013. Informed consent was taken from the subjects. Blood samples were drawn from cephalic vein in anticoagulant EDTA vacutainers (BD, USA).

### DNA Extraction

DNA was extracted from salting out method.^7^ Extracted DNA was quantified using UV spectrophotometer and integrity of DNA was checked by resolving 3 μl genomic DNA samples on 0.8% agarose gel in horizontal gel system (Bio Rad, California).

### PCR Analysis

**Fig.1:**
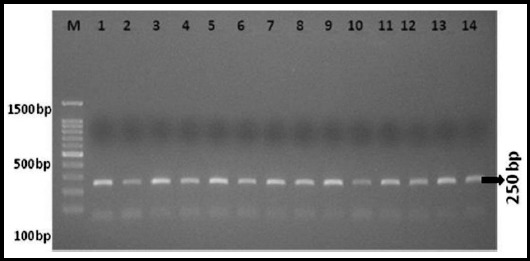
PCR analysis of CAT gene -21 A/T (rs 7943316) **M:** 100bp DNA Marker, lanes 1 to 14 PCR products

PCR was performed to amplify *CAT -21A/T (rs7943316)* gene region.^8^ The following primers were designed using online software (www.ensemble.org; www.simgene.com/primer3). Primers were purchased from Penicon (UK) for the amplification of required region of CAT gene. Forward primer 5′-AATCAGAAGGCAGTCCTCCC-3′ and the reverse primer 5′-TCGGGGAGCACAGAGTGTAC-3′ were used for the study. For PCR, total 50 μl volume was prepared containing 150 ng of genomic DNA, 0.2 mM dNTPs, 1X PCR buffer of pH-8.3, 1.5 mM MgCl_2_, 5 units of Taq DNA polymerase and PCR amplification was carried out with initial genomic DNA denaturation at 95°C for 5 minutes, followed by 35 cycles each of 60 minutes denaturation at 94°C, 40 seconds annealing at 62°C, and 60 seconds extension at 72°C. The final extension included five minutes at 72°C. Amplified PCR products of 250 bp were resolved on 1.2% of agarose gel and visualized by gel documentation system (Fig.1).

### Restriction Enzyme Analysis

PCR products were purified and treated with 10 U of *Hinf1* enzyme restriction (Thermo Scientific, Germany) and incubated at 37<°C for 16 hours. The digested products were separated on 2% agarose gel stained with visualaNA (Molequle-on, New Zealand). Gel documentation system was used for the visualization and analysis of digested PCR product.

### Sequencing of CAT (-21 A/T) gene variant

DNA sequencing was performed for the confirmation of amplified *CAT* gene product. PCR products were purified by column purification kit for DNA sequencing (www.molecule-on.com). Sequences were then subjected to perform the BLAST N analysis for confirmation of homology sequence of the amplified product.

## RESULTS

**Fig.2:**
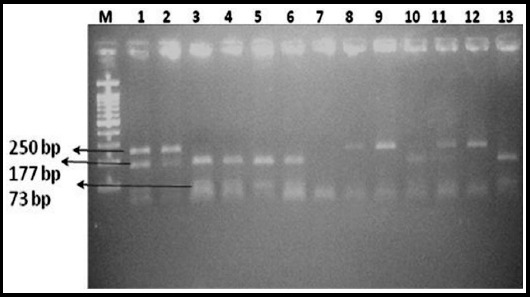
Electrophoresis band pattern of RFLP products digested by Hinf 1. **M:** 100 DNA Marker, lanes 1,2,10 and 11 heterozygous **AT:** lanes 3, 4, 5, 6 and 13 homozygous **AA:** lanes 8. 9 and 12 homozygous TT

The *CAT -21 A/T (rs7943316)* revealed the substitution polymorphism from nucleotide A to T at promoter region. It carried two allelic and three genotypic forms. Allele A is the wild type and allele T is the mutant type. PCR-RFLP investigation showed that three genotypes exist in *-21A/T (rs7943316)* polymorphism of *CAT* gene. These were homozygous AA, heterozygous AT and homozygous TT genotypes. Wild type AA genotype showed the presence of restriction site for *Hinf I* restriction enzyme and digested into two fragments of 177 bp and 73 bp. Mutant T genotype lost the restriction site for *Hinf I* and remained uncut and showed 250 bp fragments. However, heterozygous AT genotype showed all three fragments of 250 bp, 177 bp and 73 bp on 2 % agarose gel (Fig.2). It was observed that mutant T allele was found more frequently as compared to wild type A allele (Table-I). BLAST N online tool was used for this analysis of *CAT* gene sequences. After BLAST N analysis 99% homology was observed. The given table is showing the differences in T allele distribution of *CAT* gene among different countries (Table-II).

**Table I:** *CAT* *gene* polymorphism -21 A/T genotypic and allelic frequencies in healthy individuals.

*Genotypes (-21 A/T)*	*Healthy Individuals (*n* = 110)*
AA	31 (18.26%)
AT	32 (48.94 %)
TT	47 (32.8 %)
***Alleles***	
A	0.43
T	0.57

**Table-II:** Distribution of CAT gene -21 A/T mutant T allele in healthy individuals.

*Country*	*Subjects (n)*	*Genotype frequency*			*T-Allele Frequency*

		*A/A*	*A/T*	*T/T*	
Pakistan	110	31	32	47	0.57
Iran (8)	120	18	70	32	0.55
India (14)	100	7	23	70	0.82
Brazil (15)	125	23	53	49	0.60
Finland (16)	245	43	117	84	0.60
Brazil (17)	135	31	52	52	0.57
Czech Republic (18)	180	67	86	27	0.40
China (19)	848	274	472	104	0.40
China (20)	386	207	148	31	0.30
New Zealand (European) (21)	100	48	42	10	0.31

## DISCUSSION

CAT enzyme has ability to control oxidative stress by the degradation of hydrogen peroxide.^4^ Polymorphism in the promoter region of *CAT* gene may decrease the gene expression which ultimately reduce the enzymatic activity and increase the oxidative stress.^9^ In this study, frequency of mutant T allele was showing similarity with Iranian, Brazilian and Finnish populations (Table-II).

This study demonstrates *CAT -21A/T (rs7943316*) gene polymorphism in different countries. Different studies have showed the role of *CAT* gene promoter polymorphism in disease susceptibility, especially diseases related to metabolic disorders.^2^,^3^,^4^,^9^ *CAT -21A/T (rs7943316)* gene TT genotype was observed in 80% of T2DM patients along with lower CAT enzyme activity.^10^ Cerebral stroke and hypertension patients showed the association of *CAT -21A/T (rs7943316)* gene polymorphism with increased risk of disease pathogenesis.^9^ Other studies reported no association of *CAT -21A/T (rs7943316)* gene variations with insulin dependent diabetes in Czech Republic population and cardiovascular diseases in Finnish population.^4^

The present data explains prevalence of alleles and genotypes frequency of *CAT -21A/T (rs7943316)* gene polymorphism in healthy individuals. The frequency of mutant T allele may be a valuable predictor for an individual to develop disease condition. The genetic based data may facilitate new ways for early diagnosis and therapeutic interventions for diseases.

### Authors’ Contributions

**SNN** conceived and designed the experiments.

**SNN and SZ** performed the experiments and analyzed the data.

**AF** provided the blood samples and revised the initial manuscript.

**AA** coordinated the study and helped to draft the manuscript.

Each of the authors reviewed and revised the manuscript, and all authors approved the final manuscript as submitted.
